# Determinants of Skilled Birth Attendant Utilization at Chelia District, West Ethiopia

**DOI:** 10.1155/2020/9861096

**Published:** 2020-04-28

**Authors:** Adugna Dufera, Elias Teferi Bala, Habtamu Oljira Desta, Kefyalew Taye

**Affiliations:** ^1^Department of General Public Health, College of Medicine and Health Science, Ambo University, Ambo, West Shoa, Ethiopia; ^2^Department of Public Health College of Medicine and Health Science, Ambo University, Ambo, Ethiopia P.O. Box 19

## Abstract

**Background:**

An estimated 303,000 maternal deaths occurred globally in 2015 from which sub-Saharan Africa alone accounted for 201,000 (66%) of the maternal deaths, and most of these are attributed to complications of pregnancy and childbirth due to the absence of institutional delivery by skilled attendants.

**Objective:**

The aim of this study was to assess institutional delivery utilization and associated factors among mothers who gave birth in the last one year in Chelia District. *Methodology*. A community-based cross-sectional study design supplemented by a qualitative method was employed from March 15 to 30, 2018. A multistage sampling technique was used to select 475 study participants. Quantitative data were collected using structured questionnaires, and focus group discussions were employed to get qualitative data. The data were entered to EpiData version 3.1 and exported to the statistical package version 21 for analysis. Descriptive statistics and bivariate and multivariate logistic regression analysis were computed to measure the strength of association between dependent and independent variables at a *p* value of <0.05.

**Results:**

Among the respondents, 216 (46.2%) utilized institutional delivery service. Monthly income (AOR = 4.465, 95%CI = 1.729, 11.527), antenatal care attendance (AOR = 0.077, 95%CI = 0.008, 0.73), knowledge of mothers about their expected date of delivery (AOR = 0.297, 95%CI = 0.179, 4.93), intended pregnancy (AOR = 0.326, 95%CI = 0.162, 0.654), discussion with health extension workers about the place of delivery at home visit (AOR = 0.11, 95%CI = 0.023, 0.523), knowledge of mothers about the existence of the waiting area in health facilities (AOR = 0.14, 95%CI = 0.023, 0.84), and number of children (AOR = 0.119, 95%CI = 0.029, 0.485) had a significant association with institutional delivery utilization.

**Conclusion:**

Utilization of institutional delivery was low and far away from the expected country target in the district. The health sector should strive to increase proportion of institutional delivery by reaching pregnant mothers with timely antenatal care service provision and enhancing family planning provision.

## 1. Introduction

The majority of maternal health complications and deaths occurred in low- and middle-income countries (LMIC) where three quarters of the deaths are due to direct obstetric complication [1, 2]. Although maternal deaths worldwide have decreased by 45% since 1990, 800 women still die each day from largely preventable causes before, during, and after the time of giving birth. Ninety-nine percent of the preventable maternal deaths occur in low- and middle-income countries [3].

An estimated 295,000 maternal deaths occurred globally in 2017, making an overall MMR of 211 maternal deaths per 100,000 live births. Out of this, least developed countries account for a maternal mortality rate of 415 per 100,000 live births, with sub-Saharan Africa being the only region with very high MMR 542 per 100,000 live births and Ethiopia accounting for 14,000 maternal deaths [4, 5].

It is indicated that the risk of dying from a maternal cause for a woman in developing countries especially in Africa is around 23 times higher than that for a woman living in developed countries and the maternal mortality ratios are 495 and 12 per 100,000 women in Africa and in developed regions, respectively [6, 7].

From the recent Ethiopian Demographic and Health Survey report, the estimate of the maternal mortality ratio was 412 deaths per 100,000 live births, which could be prevented through skilled personnel intervention for all maternal services particularly before, during, and after childbirth [8].

Appropriate delivery care is crucial for both maternal and prenatal health, and increasing skilled attendants at birth is the central goal of the safe motherhood and child survival movements. In addition to professional attention, it is important that mothers deliver their babies in an appropriate setting where lifesaving equipment and hygienic conditions can also help reduce the risk of complications that may cause death or illness to mother and child [4, 9].

When a woman dies in childbirth, her infant and any other children's survival is threatened. Infants without a mother are more likely to die within two years. Children up to 10 years whose mothers die are 3 to 10 times more likely to die within two years than children living with mothers [10, 11].

Literatures recommend that the best way to assure a safe and successful delivery outcome remains to be ensuring the presence of a skilled birth attendant in every childbirth [12, 13]. This can be realized by increasing proportions of institutional deliveries [14].

Home delivery is common in many developing countries including Ethiopia. A high rate of home delivery was reported from Malawi, Nepal, Zaria (Northern Nigeria), and Pakistan [2, 7, 15], and many studies in Ethiopia, such as in Tigray, Arsi, and Dodota districts, indicated low utilization of institutional delivery. Similarly, the 2016 EDHS report revealed that the majority of births were being attended by traditional birth attendants at home [8, 15–17].

Different studies have indicated that institutional delivery service utilization is determined by complex interacting factors such as level of need, distance, economic factors, awareness and satisfaction, sociodemographic characteristics, and administrative arrangements [12, 13, 18].

Beside importance of institutional delivery, there was very limited evidence regarding the raised issues. Therefore, this study was aimed at investigating the level of institutional delivery service utilization and factors associated with it in Chelia District, western part of Ethiopia. The findings may be of immediate benefit to the West Shoa Zone health department in the implementation of future conducive strategies aimed at modifying factors in the health system in the community as agents of change to enhance institutional delivery.

## 2. Methods and Materials

### 2.1. Study Area and Design

The study was conducted in Chelia District West Shoa Zone, Oromiya regional state, Ethiopia. The district has 18 rural and 2 urban kebeles (kebele refers to the lowest administrative unit in Ethiopia). The total population of the district is 108,886 out of which women of childbearing age and expected pregnancies were 24,096 and 3,778, respectively. On the other hand, in the district, there were one general hospital, four health centers, eighteen health posts, and nine private clinics. The study was conducted starting from March 15 to 30, 2018. A community-based cross-sectional study design supplemented by a qualitative study was utilized.

### 2.2. Study Populations and Sample Size

All women of the reproductive age group were the source population for this study. Randomly selected women who gave birth in the last 12 months were included in the study. The following assumptions were considered while calculating the sample size. Ninety-five percent confidence level (95% CI) of obtaining mothers who gave birth at the health institutions from the population within 5% margin of error and using *p* = 16.9% institutional delivery prevalence of Gonji Kolela District of Amhara Region, Ethiopia [12].

(1) $$\begin{array}{c} n=\frac{{\left(Z\alpha /2\right)}^{2}\left(P\right)\;\left(1-p\right)\;}{d^{2}}=\frac{{\left(1.96\right)}^{2}\left(0.169\right)\;\left(1-0.169\right)\;}{0.05^{2}}=216. \end{array}$$

Considering the design effect of 2 and 10% nonresponse rate, the final sample size was 475.

### 2.3. Sampling Technique

The multistage sampling technique was used to select the study participants. Six kebeles (one urban and five rural) which account for 30% of the total kebeles were selected from each stratum using a lottery method, and 475 study participants were selected from each selected kebele proportionally using systematic sampling methods. The first HH was sampled using the lottery method by going at the health post of the kebele, and the sampling interval was calculated as *K* = (*N*/*n*) = 1179/475~3. Thus, households were selected by using a systematic sampling procedure in an interval of every 3^rd^ households in each kebele. Since mothers in the specific kebeles were expected to be homogenous in many ways, convenient sampling was employed for FGD to select easily accessible mothers for the discussion. Five FGDs having 6 to 8 participants were employed.

### 2.4. Data Collection Tool

#### 2.4.1. Quantitative Data

A structured questionnaire which included sociodemographic and health service utilization factors was prepared based on the objectives of the study after reviewing different literatures. Then, the questionnaire was translated to the local language to facilitate the data collection process.

#### 2.4.2. Qualitative Data

To have an in-depth insight into the factors related to the place of delivery, qualitative data was collected. Five focus group discussions were held in the district. Three groups consisted of mothers, and the remaining two groups were husbands. Each FGD had 6 to 8 participants. Each session took a time span of one to one and half hour. The groups were made homogeneous in terms of sex and social classes. Settings for the discussions were arranged with privacy for participants, comfortable situation (location), lesser noise (by avoiding noisy areas), nonthreatening environment, and easily accessible location. The recruited supervisors were used as the facilitator and note taker while the principal investigator has modulated the discussion.

### 2.5. Data Processing and Analysis

#### 2.5.1. Quantitative Data

Data was coded and entered to EpiData version 3.1 and exported to SPSS version 21 for analyses. Frequency, mean, and standard deviations from descriptive statistics and analytic statistics such as bivariate and multivariable logistic regression analyses were computed to determine the association of various factors on the outcome variable. Variables having a *p* value less than or equal to 0.05 on binary logistic regression were the candidate for multiple logistic regressions. Statistical significance was declared at *p* < 0.05. The strength of association between independent and dependent variables was assessed using the adjusted odds ratio with 95% confidence interval.

#### 2.5.2. Qualitative Data

Data was transcribed into Afaan Oromo text by the principal investigator from replaying the recorded discussions and using the written note from ideas raised during the sessions and translated into the English language for analysis. Different ideas in the text were merged in their respective thematic areas and analyzed thematically. Then, the result was presented in narration by triangulating with the quantitative findings.

### 2.6. Ethical Consideration

Ethical clearance was received from the Ethical Review Committee of College of Medicine and Health Science of Ambo University on February 28, 2018, with reference no. CMHS-ERC: 28/2010. Verbal consent was obtained from the study subjects after they had been informed about the objectives and procedures of the study including their right to withdraw themselves from participation any time they want.

## 3. Results

### 3.1. Sociodemographic Characteristics

A total of 468 mothers who gave birth in the last twelve months were interviewed making a response rate of 98.5%. Out of the interviewed mothers, 396 (84.6%) were rural dwellers and 161 (34.4%) were in the age category of 25-29 years. The mean age of the respondents was 28.85 years with the standard deviation (SD) of 5.58. Most of the respondents 302 (64.53%) were Protestant in religion whereas majority of them 427 (91.24%) were Oromo in ethnicity. The mean monthly income of the respondents was 1221.18 ETB with a standard deviation of 1632.94 (Table 1).

### 3.2. Obstetric Characteristics of the Respondents

During the last pregnancy, 395 (84.4%) of the mothers had attended ANC visit. Among the mothers who attended ANC, more than half 237 (60%) visited health facilities less than four times. About 164 (35%) of the respondents faced more than five pregnancies including the current (Table 2).

### 3.3. Institutional Delivery Service Utilization

**Figure 1:**
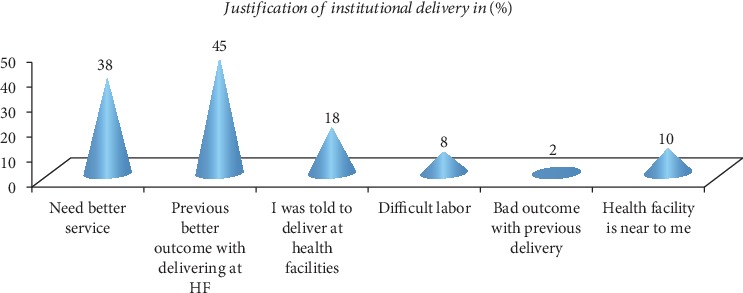
Justifications given by mothers who utilized institutional delivery in Chelia District (March 2018).

Among the study participants, only 216 (46.2%) gave births at health institutions and the rest delivered at home. Mothers who gave birth at health facilities justified what made them go to health institution to give birth. The points raised as reasons for their institutional delivery were as follows: previous better outcome with delivering at health facility, need better services, I was told to deliver at health facility, and health facility was near to me (Figure 1).

### 3.4. Factors Affecting Institutional Delivery Service Utilization

Multivariate analysis showed that monthly income, ANC attendance during the last pregnancy, EDD information, intended pregnancy, discussion with HEWs about the place of delivery, knowledge of mothers about the existence of the waiting area in health facilities, and number of children had a significant statistical association with the utilization of institutional delivery service (see Table 3).

Mothers who have six or more children were about 88.1% (AOR = 0.119, 95%CI = 0.029, 0.485) less likely to utilize institutional delivery as compared to those who have three or less children. In addition, mothers who did not get discussion with HEWs about the place of delivery were 89% (AOR = 0.11, 95%CI = 0.023, 0.523) less likely to utilize institutional delivery than those who got the discussion. Similarly, mothers who become pregnant without their need were 67.4% (AOR = 0.326, 95%CI = 0.162, 0.654) less likely to utilize institutional delivery as compared to those who got intended pregnancy. Furthermore, mothers who did not attend ANC during the last pregnancy were 92.3% less likely to give birth at health institutions than those who attended ANC (AOR = 0.077, 95%CI = 0.008, 0.73). Finally, this study also showed that having knowledge of EDD increases the likelihood of giving birth at health institutions. Mothers who did not know their EDD during pregnancy were 70.3% less likely to utilize institutional delivery than those who knew it (AOR = 0.297, 95%CI = 0.179, 4.93) (Table 3).

Qualitative data from FGD also showed that income affects institutional delivery service utilization. A FGD participant spoke that “…there is difference between poor and riches in utilizing institutional delivery; even in the absence of ambulance, using local ambulance was challenge in that no one carries pregnant of poor family. In the presence of money sky is a way” (a 47-year-old participant's husband who has 8 children).

A 36-year-old rural dweller mother of 6 children said “…Giving normal birth is not in the hand of peoples; it is just from God. I believe that God is with me at where ever I go; so, no need of going here and there.”

A mother aged 34 years added that “...let ears of pregnant do not hear this; I know many mothers who died in Hospitals, no one died at home to give birth. What God determined for us could not be left at everywhere.”

## 4. Discussion

The results of this study showed that mothers who utilized institutional delivery service were 46.2% (95% CI: 41.8, 50.9) in the district and more than half of the mothers (53.8%) gave birth at home. This magnitude of institutional delivery was low when compared with the health sector development plan of the country. However, it is higher than those in studies conducted in Gonji Kolela District of Amhara Region, Ethiopia; Dodota District of Oromiya, Ethiopia; and Sekela District of Amhara, Ethiopia, which were 16.9%, 18.2%, and 12.1%, respectively [6, 10, 12, 13]. Similarly, this finding was higher than that of the 2016 EDHS report of the national and Oromiya region which were 26% and 18.8%, respectively [12, 19, 20]. The difference may be due to the study period, geography of the health facility, and existence of free ambulance service. For instance, if free ambulance service is given, pregnant mothers will reach health institutions without transport expense. On the other hand, the finding of this study was lower than those of studies conducted in Dejen District, Ethiopia, and Bench Maji Zone, southern Ethiopia, which were 71.7% and 78.3%, respectively [13, 14]. These differences may be due to the study period and area in which health facilities could be easily accessible and sustainable ambulance services might be in place.

The result of this study revealed that income had a significant association with institutional delivery service utilization. Mothers who earn beyond 1,500 Ethiopian birr were 4.46 times more likely to give birth at health facility as compared to those who earn less than 500 Ethiopian birr. This finding was consistent with other research findings reported in northern Karnataka of India and Gonji Kolela District of Amhara [9, 13, 21]. The possible explanation for this consistency could be the importance of money in life facilitation for human beings regardless of the residence area.

ANC attendance during the last pregnancy was also another determinant factor of institutional delivery. Mothers who did not attend ANC during the last pregnancy were 92.3% less likely to utilize institutional delivery service as compared to those who attended ANC. This finding was similar to the findings from studies conducted in Goba District of Oromiya of Ethiopia and Tigray of Ethiopia [22, 23]. ANC service is a contact point for health workers with pregnant mothers to promote an institutional delivery and provide information for mothers on the status of their pregnancy which in turn alerts them to decide where to give birth. This finding was supported also by qualitative data.

A mother who completed grade 12 participated in FGD and also raised that “giving birth in Health facility is important. It was God who gave this wisdom for health professionals. Utilizing the services is not sin. Many mothers start services during pregnancy, but some do this only to know sex of the unborn. I and my neighbor attended the service and we got advice about our place of delivery from health professionals during the visits. Finally, both of us gave birth at the facility.”

The results of this study showed also that institutional delivery service utilization was significantly influenced by the number of children the respondents have. Women who possess more than five children were about 88.1% less likely to deliver at health facilities than those who possess three or less children. This may be due to the fact that their previously experienced home delivery may make them confident enough to be riskless for the next childbirth among those who have more children.

In the presence of a home visit program by HEWs, the existence of discussion about the place of delivery with pregnant women was significantly associated with institutional delivery service utilization. Mothers who have not discussed with HEWs regarding the delivery place were 89% less likely to utilize institutional delivery service as compared to those who had the discussion. This may be because of awareness gained when family-based discussion with HEWs takes place during home visit.

Qualitative finding also showed that information from health extension workers is crucial for the health of the community. A 47-year-old FGD discussant father of 8 children said that “formerly health extension workers visit our home and give health information for our women, but currently they simply earn salary; they do not know us, we do not know them.”

Not only is ANC service during pregnancy important for medical checkup and risk assessment but it also exposes the pregnant mothers to useful health information like eating habit, their EDD, and delivery preparation. This study found that EDD information is another significant factor for institutional delivery service utilization. Mothers who had not been informed about their EDD during pregnancy were 70.3% less likely to utilize institutional delivery service than those who knew their EDD. If delivery appointment is approximately predicted and known, mothers may prepare themselves accordingly by making some necessary arrangements for the delivery. This may keep the mothers alert and help them utilize institutional delivery service.

One of the institutional delivery service-promoting factors that recently started in public health facilities of our country is a waiting area with home-like environment for laboring mothers. This factor was significantly associated with institutional delivery service utilization in this study. Mothers who did not know that a home-like waiting area was available in health facilities were 86% less likely to utilize institutional delivery service as compared to those who knew it. Promoting this waiting area encompasses some food services such as porridge and coffee which were considered common food for mothers who gave birth. This may attract mothers to be in health facilities before and after birth just as their home. Qualitative findings also revealed significance of this factor.

As qualitative data indicated, hospitality and sympathy of health care providers determine the health-seeking behavior of clients. A 27-year-old FGD discussant mother of 2 children stated that “I have delivered in health facility; before that I went there with my aunt. We faced a compassionate and respectful attendant; she approached us as our sister and helped our aunt to give normal birth. There was coffee ceremony during that night. This makes me give birth at Health facility.”

The study revealed also that intent of mothers about pregnancy was also significantly associated with the institutional delivery service utilization. Mothers who got accidental pregnancy were 67.4% less likely to utilize institutional delivery service as compared to those who got pregnant intentionally. Intended pregnancy makes the family eagerly ready for birth of the new born, and this may create conducive conditions for the mothers to deliver at health facilities.

## 5. Conclusion and Recommendation

This study revealed that proportion of women utilizing institutional delivery service in the study area was still low as compared to national and regional target of the country. Antenatal care attendance, family income, number of children, expected date of delivery information, intended pregnancy, existence of the delivery place related to the discussion between HEWs and mothers during home visit, and knowledge of the existence of home-like environment in public health facilities were factors affecting institutional delivery utilization of women in the district.

Since institutional delivery is vital for the well-being of both mothers and newborns, it is recommended to be enhanced throughout the district. Health extension workers should strengthen home visits focusing on households with pregnant mothers to expose them to health information and antenatal care services. Antenatal care service providers also should tell each pregnant mother about their expected date of delivery and the existence of respective caregivers with full-package waiting areas in the facility. Unintended pregnancy and a high number of children should be minimized through family planning service provision since they in turn affect institutional delivery service utilization. Pregnant mothers should visit health institutions as early as possible so as to get necessary services and health information prior to their delivery time.

The District Health Office should strive to enhance a pregnant registration system in the community, promoting conference of pregnant mothers with midwives or health extension workers and antenatal care service provision strategies for all eligible mothers through strong and continued monitoring of the work of health extension workers throughout the district so that institutional delivery utilization will be improved. The District Health Office should also have a discussion forum with traditional birth attendants and other home delivery assistants so that they should promote institutional delivery by skilled attendants rather than assisting at home.

## Figures and Tables

**Table 1:** Sociodemographic characteristics of mothers who gave birth in the last one year in Chelia District, western Ethiopia (March 2018) (*N* = 468).

Variables	Categories	Frequency	Percent
Age of the respondents	≤20	37	7.9
	20-24	60	12.8
	25-29	161	34.4
	30-34	126	26.9
	>35	84	18

Religion of the respondents	Orthodox	149	31.84
	Muslim	3	0.64
	Wakefata	14	2.99
	Protestant	302	64.53

Ethnicity of the respondents	Oromo	427	91.24
	Amhara	22	4.70
	Tigre	1	0.21
	Gurage	18	3.85

Marital status of the respondents	Single	20	4.27
	Married	428	91.45
	Divorced	14	3
	Widowed	6	1.28

Educational status of the husbands	Unable to read and write	113	26.40
	Primary school	161	37.62
	Secondary school	96	22.43
	College and above	58	13.55

Educational status of the respondents	Unable to read and write	176	37.6
	Primary school	179	38.2
	Secondary school	70	15
	College and above	43	9.2

Occupation of the respondents	Government or NGO employee	42	8.97
	Merchant	33	7.05
	Housewife	51	10.90
	Daily labor	28	5.98
	Private employee	65	13.89
	Farmer	244	52.14
	Unemployed	5	1.07

Residence of the respondents	Rural	396	84.6
	Urban	72	15.4

Access to information sources like radio or television	Yes	168	35.9
	No	300	64.1

Average monthly income of the respondents	≤500	154	32.91
	501-1000	178	38.03
	1001-1500	53	11.32
	>1500	83	17.74

**Table 2:** Obstetric characteristics of mothers who gave birth in the last one year in Chelia District, western Ethiopia (March 2018) (*n* = 468).

Variables	Alternatives	Frequency	Percent
Number of children	≤3	234	50.0
	4-5	137	29.3
	≥6	97	20.7

Gravidity	≤2	134	28.6
	3-4	154	32.9
	≥5	180	38.5

Parity	≤2	151	32.3
	3-4	153	32.7
	≥5	164	35.0

Status of the new born	Alive	454	97.0
	Other^∗^	14	3

Home delivery assistant	Family/neighbors/friends	153	61
	Health extension workers	25	10
	TBAs	56	22
	None	18	7

Knowledge of pregnancy danger signs	Yes	199	42.5
	No	269	57.5

ANC attendance	Yes	395	84.4
	No	73	15.6

ANC started time in gestational weeks	≤12 weeks	126	31.9
	13-24	241	61.01
	>24	28	7.09

Number of ANC visits	One	7	1.77
	Two	73	18.48
	Three	157	39.75
	Four	95	24.05
	Beyond 4	63	15.95

Knowledge about EDD	Yes	189	47.85
	No	206	52.15

Delivery site	Home	252	53.8
	Health facility	216	46.2

∗ indicates still birth or death within seven days.

**Table 3:** Factors associated with institutional delivery service utilization of mothers who gave birth in the last one year in Chelia District, western Ethiopia (March 2018) (*n* = 468).

Variables	Alternatives	Delivery sites		COR (95% CI)	AOR (95% CI)
		HF	Home		
Monthly income	≤500	41	113	1	1
	501-1000	82	96	2.35 (1.48, 3.74)^∗∗^	3.64 (1.63, 8.15)^∗∗^
	1001-1500	28	25	3.087 (1.61, 5.89)^∗∗^	3.01 (1.106, 8.152)^∗^
	>1500	65	18	9.95 (5.28, 18.73)^∗∗^	4.46 (1.72, 11.52)^∗∗^

Number of children	≤3	130	104	1	
	4-5	54	83	0.52 (0.33, 0.79)^∗∗^	0.39 (0.142, 1.09)
	≥6	32	65	0.39 (0.24, 0.646)^∗∗^	0.119 (0.029, 0.48)^∗∗^

Residence of the respondents	Rural	162	234	0.23 (0.131, 0.40)^∗∗^	5.74 (0.9, 36.59)
	Urban	54	18	1	1

HEWs ever visit home	Yes	78	72	1	1
	No	138	180	0.708 (0.47, 1.04)	

Discussion with HEWs about the delivery place	Yes	72	56	1	1
	No	6	16	0.29 (0.107, 0.79)^∗^	0.11 (0.02, 0.52)^∗∗^

ANC attendance	Yes	211	184	1	1
	No	5	68	0.06 (0.025, 0.16)^∗∗^	0.07 (0.008, 0.73)^∗^

Knowledge about EDD	Yes	138	51	1	1
	No	73	133	0.20 (0.132, 0.31)^∗∗^	0.29 (0.179, 4.93)^∗∗^

Intended pregnancy	Yes	195	172	1	1
	No	21	80	0.23 (0.137, 0.39)^∗∗^	0.32 (0.162, 0.65)^∗∗^

Knowledge about the existence of waiting areas in public HF	Yes	182	161	1	1
	No	34	91	0.33 (0.21, 0.517)^∗∗^	0.14 (0.02, 0.84)^∗^

^∗^ *p* value < 0.05, ^∗∗^*p* value < 0.01. HF = health facilities.

## Data Availability

The data used for this study are available from the corresponding author when demanded.

## Abbreviations

ANC: Antenatal care
EDD: Expected date of delivery
EDHS: Ethiopian Demographic and Health Survey
EPMM: Ending preventable maternal mortality
FGDs: Focus group discussions
HEWs: Health extension workers
HF: Health facility
SDGs: Sustainable development goals
HSTP: Health sector transformational plan
TBAs: Traditional birth attendants
SBAs: Skilled birth attendants.
